# Fully endoscopic far-lateral supracerebellar infratentorial approach for trigeminal neuralgia: illustrative case reports

**DOI:** 10.1186/s41016-023-00353-0

**Published:** 2024-01-02

**Authors:** Hong Yao, Wenlei Yang, Hao Tang, Yijun Cheng, Shaojian Lin, Zhe Bao Wu

**Affiliations:** Department of Neurosurgery, Center of Pituitary Tumor, Ruijin Hospital, Shanghai Jiao Tong University School of Medicine, 197# Ruijin Er Road, Shanghai, 200025 China

**Keywords:** Approach, Endoscopic surgery, Trigeminal neuralgia

## Abstract

**Background:**

Trigeminal neuralgia (TN) is a common cause of craniofacial pain. The retrosigmoid approach is usually used to treat TN, but no cases of endoscopic far-lateral supracerebellar infratentorial approach (EF-SCITA) were used to undergo operation for TN.

**Case presentation:**

Two patients were presented with severe facial pain and preliminary diagnosis was TN. Preoperative magnetic resonance imaging revealed that a superior cerebellar artery (SCA) compressed the trigeminal nerve in case 1, and a tumor located in the petrous apex extending into the Meckel’s cave compressed the trigeminal nerve in case 2. Operations were achieved through the EF-SCITA. The pain was totally relieved with no postsurgical complications in both cases.

**Conclusions:**

We present the first two case reports of EF-SCITA to relieve classical and secondary TN successfully. The EF-SCITA can be a promising approach for treating TN.

## Background

Trigeminal neuralgia (TN) is an exemplary neurological condition with severe, stimulus-evoked, stabbing pain attacks in the face [1]. According to the third edition of the International Classification of Headache Disorders, TN is classified into classical TN, secondary TN, and idiopathic TN [2]. Classical TN requires demonstration of vascular compression with morphological changes of the trigeminal nerve root on magnetic resonance imaging (MRI) or during surgery [2]. Secondary TN is due to an identifiable underlying neurologic disease, such as tumors or multiple sclerosis [2]. Idiopathic TN occurs with unknown causes [2]. Neurophysiologic tests and imaging that establish the etiology of classical or secondary TN determine definite neuropathic facial pain that occurs in the distribution of the trigeminal nerve branches [1, 3]. Patients with vessel-related or tumor-related TN are often subject to neurosurgical intervention.

Microvascular decompression (MVD) via the retrosigmoid approach is the first-choice surgery in patients with classical TN [4, 5]. The purely endoscopic assisted MVD may be becoming a future trend, because it affords surgeons a better intraoperative view, an equally good operational outcome for the patients with lower complication rates than a traditional microscopic MVD [5]. For tumor-induced TN, tumor resection surgery with nerve decompression is considered to be a first-line therapy [6]. The choice of operation approach is variable determined by the nature of the pathology and location relative to key structures and extension into surrounding structures, including commonly used retrosigmoid approach. Herein, we report a novel fully endoscopic far-lateral supracerebellar infratentorial approach (EF-SCITA) for classical or secondary TN.

## Case presentation

### Case 1

**Fig. 1:**
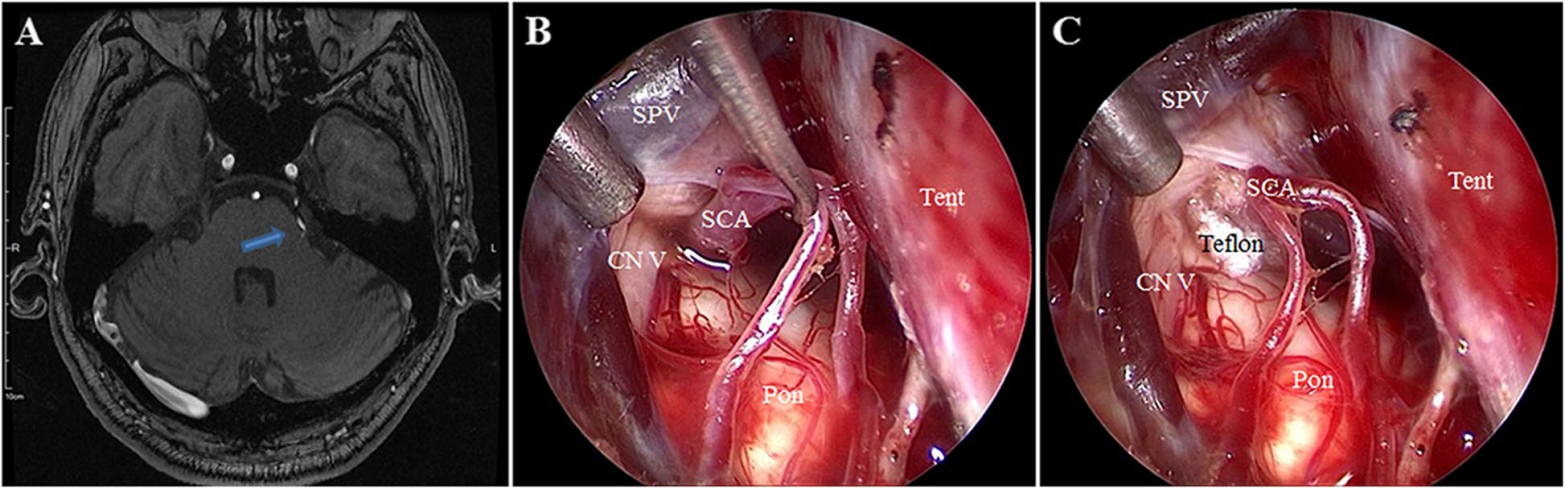
Preoperative and intraoperative findings in case 1. **A** Preoperative head magnetic resonance imaging of the patient in case 1. **B** Under the endoscopic view, the trigeminal nerve was compressed into an angle by the SCA in the root entry zone. **C** The Teflon pads were placed between the trigeminal nerve and the SCA under the endoscopic view. The blue arrow indicated the trigeminal nerve compression by the offending vessel. SPV, superior petrosal vein; SCA, superior cerebellar artery; CN V, trigeminal nerve; Ten, tentorium; Pon, Pons

A 73-year-old male was admitted to our hospital. His main clinical symptom was paroxysmal severe pain for decades of seconds in the left face for approximately 2 years that was induced by chewing and brushing teeth. The physical examination was normal and the pathologic signs were negative. All laboratory tests, including routine blood, biochemical parameters, liver and kidney function, and blood coagulation function tests were within normal limits. The patient was diagnosed with left trigeminal neuralgia and was treated with carbamazepine, but the effect was not satisfactory. Preoperative MRI of the head showed that the left superior cerebellar artery (SCA) compressed the root entry zone of the left fifth cranial nerve (Fig. 1A). EF-SCITA was ultimately chosen and designed as follows. After operation, the pain was relief immediately. There were no postoperative complications and no evidence of recurrence at the 6-month follow-up evaluation.

### Case 2

**Fig. 2:**
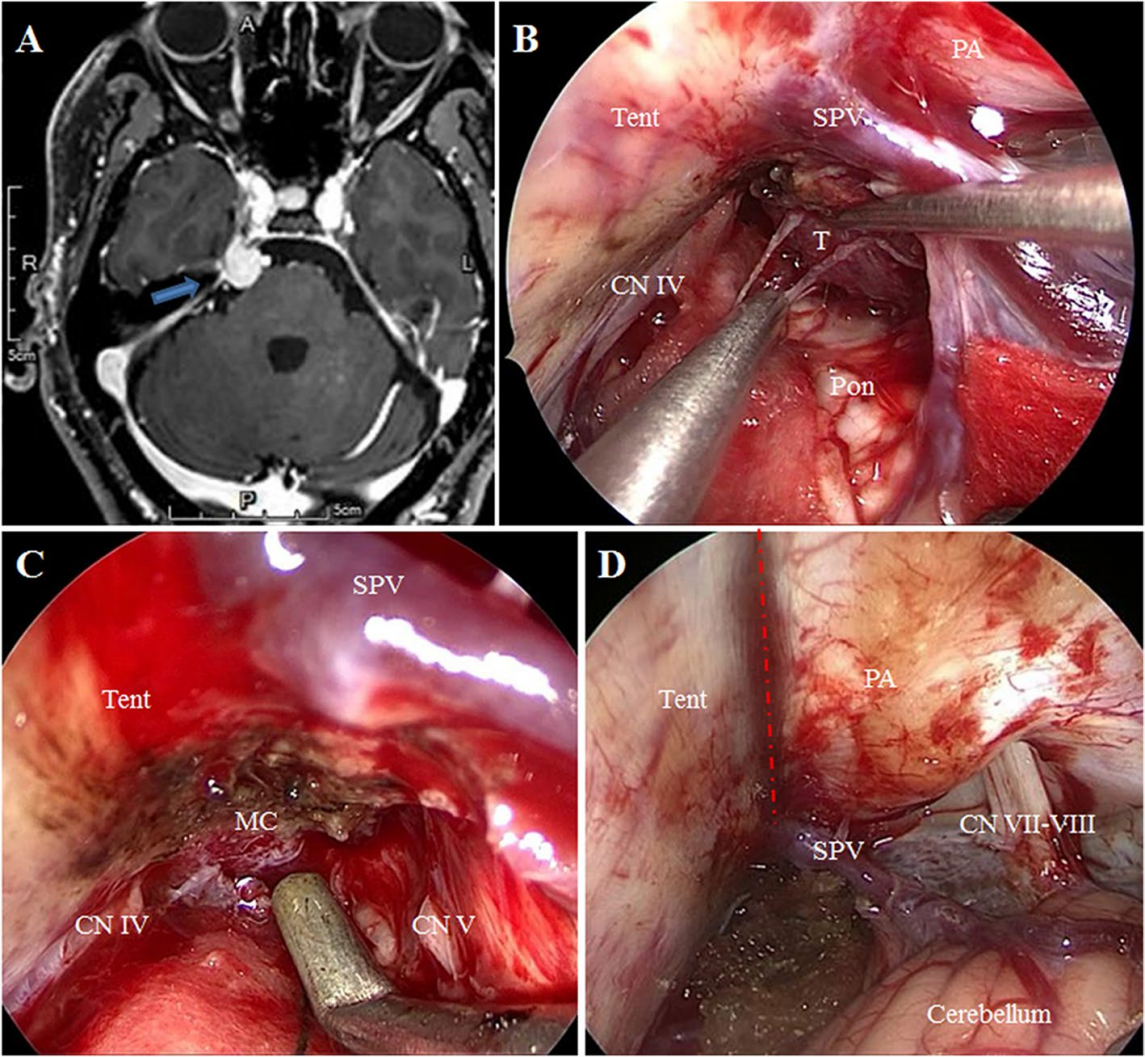
Preoperative and intraoperative findings in case 2. **A** Preoperative head magnetic resonance imaging of the patient in case 2. **B** Under the EF-SCITA view, a solid tumor was seen behind the SPV. **C** The tumor was dissected from the MC. **D** Endoscopic full view of the EF-SCITA after the operation. The blue arrow indicated the tumor in the PA extending to the MC. SPV, superior petrosal vein; SCA, superior cerebellar artery; Ten, tentorium; Pon, Pons; PA, petrous apex; MC, Meckel’ cave; CN IV, trochlear nerve; CN V, trigeminal nerve; CN VII-VIII, acoustic-facial bundle

A 54-year-old male patient presented with intermittent tearing pain in the right face that had lasted for two years, and was admitted to our hospital. He had no obvious trigger point for the pain. His physical examination revealed hypoesthesia and masticatory atrophy in the right face. His corneal reflex was present. All physical tests, including routine blood examinations, assessments of liver and kidney, and evaluation of blood coagulation functions, were within normal ranges. The patient had no unambiguous history of trauma or no family history of genetic disorders. The oral gabapentin treatment was no effect, and the pain became aggravated. Preoperative cranial MRI showed that a small tumor in the right petrous apex extending into the Meckel’s cave (Fig. 2A). Finally, the patient received fully endoscopic tumor resection via the EF-SCITA. He was significantly relieved from the pain and discharged 5 days later. There was no recurrence after 6 months of follow-up.

### Surgical procedure

**Fig. 3:**
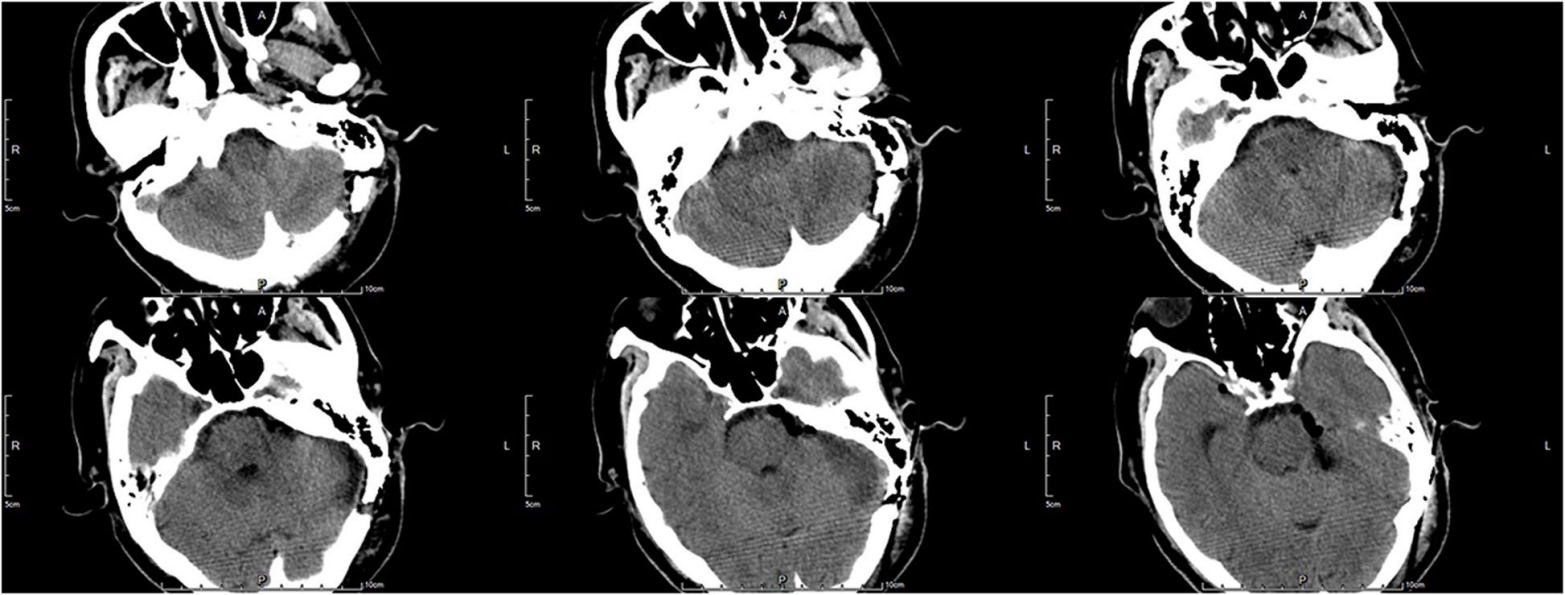
Postoperative CT images of case 1. The images showed the surgical area was clean

**Fig. 4:**
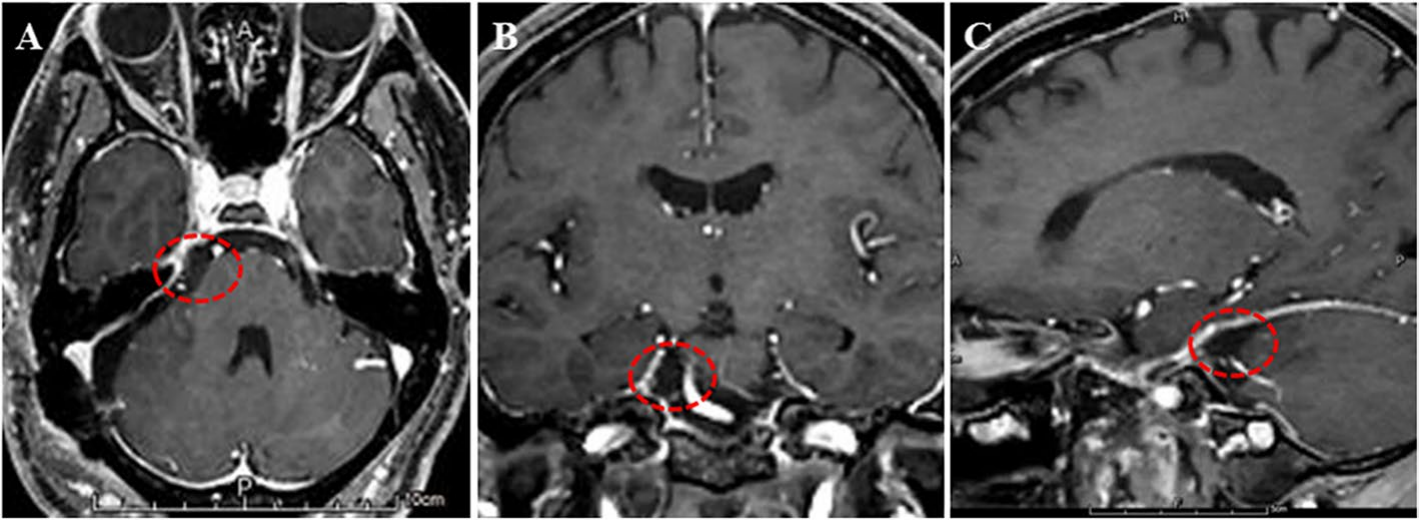
Postoperative MRI images of case 2. **A** Postoperative axial (**A**), coronal (**B**), and sagittal (**C**) enhanced head MRI of the patient in case 2 showed that the meningioma was totally removed

Both patients consented to undergo surgery via EF-SCITA. After general anesthesia, the patient was placed in a lateral position or park-bench position with the head drooping at 15° and rotated at 10° to the contralateral side. The jaw was placed about two transverse fingers away from the sternum, and the mastoid process on the operational side was at the highest position. The endoscopic monitor and endoscopic pneumatic arm holder were located on the contralateral of the operator [7]. A transverse postauricular 6-cm incision was performed. The suboccipital craniotomy of about 3 × 3 cm bone window, that can be much smaller in endoscopic MVD surgery, was conducted using a grinding drill, and the transverse and sigmoid sinuses were fully exposed. Then, the “X” shaped dural flap was cut and turned over to the margin of the transverse sinus. The arachnoid membrane of cisterna magna was cut to release cerebrospinal fluid. The upper body was elevated 30° to allow gravity retraction of the cerebellum. The infratentorial space was expanded by gravity retraction; it was easily seen that the root entry zone of the trigeminal nerve was significantly compressed by SCA in case 1 (Fig. 1B), and the tumor was seen just locating behind the superior petrosal vein (Fig. 2B) and was intraoperative pathology diagnosed as meningioma in case 2. Using 30° endoscopy, three Teflon pads were placed piece by piece between the offending vessels and the trigeminal nerve in case 1 (Fig. 1C), and the tumor extending into the Meckel’s cave was totally resected piece by piece in case 2 (Fig. 2C). Under the full view of the EF-SCITA after the operation, the cerebellopontine angle was clearly visible (Fig. 2D). The superior petrosal vein was well protected in the two cases. The dura mater was closed in a waterproof fashion, the bone flap was fixed back with titanium plates and screws, and the muscle and skin incision were sutured layer by layer. Postoperative image examinations were performed in both cases (Figs. 3 and 4).

## Discussion

For most TN, a retrosigmoid approach is sufficient to expose and treat the lesion [8, 9]. However, sometimes, the operator may pass through dense and vital cranial vessels and nerves, especially the petrosal veins lying in front of the trigeminal nerve and making the exploration very difficult. The postoperative complications may be difficult to avoid, particularly vein damage or venous infarction due to vein sacrificing, facial paresis or weakness, and hearing loss [5]. In addition, the suprameatal tubercle may obstruct the exposure of the Meckel’s cave and petrous apex region from the lateral to medial view. Drilling the obstructed suprameatal tubercle will make the operation more complex and potentially risky.

The EF-SCITA, in these two cases, was a novel approach strategy for TN. The infratentorial space, just like the nasal cavity, it is a natural space for the brain, with no important nerves or blood vessels on its channels. Combined with the sagging or traction of the cerebellum and the displacement of the transverse sinus, this space can provide sufficient exposure. Of course, for the endoscopic MVD surgery, the brain spatula is usually no used. The EF-SCITA is a new attempt. In case 1, we can observe the compression of SCA on the root exit zone of the trigeminal nerve clearly. However, for the retrosigmoid approach, the nice intraoperative vision is often difficult. After sufficient decompression, the pain immediately relieved postoperatively. In addition, for the tumor in case 2, which extend into the Meckel’ cave, the EF-SCITA can provide better vision and opportunities for total tumor resection. Compared to the subtemporal approach, the EF-SCITA has a smaller trauma and potential complication rate.

In addition, the endoscopy has gradually become a widely used technique because of its advantages in direct and angled views in China. The EF-SCITA is a booming approach, and it has been recently reported to successfully resect tumors in the petroclival region and the suprasellar region with posterior fossa extension [7, 10]. The novel approach can successfully access to the cistern and Meckel’s cave segments of the trigeminal nerve. In terms of development, this approach can also be further applicable for lesions with the middle fossa extension. Combining the advantages of the infratentorial approach and the endoscopy, we believe that EF-SCITA may bring a new round of development impetus to TN. Furthermore, long-term studies with larger samples and consistent medical parameters and controls are needed to be further observed.

## Conclusions

In the two cases, the EF-SCITA was first reported to apply to TN. All postoperative symptoms were significantly relieved with no postoperative complications, and there were no recurrence evidences in patients. Thus, the fully EF-SCITA is a relatively safe, feasible, and effective approach for TN.

## Data Availability

Not applicable.

## Abbreviations

EF-SCITA: Endoscopic far-lateral supracerebellar infratentorial approach
TN: Trigeminal neuralgia
SCA: Superior cerebellar artery
MRI: Magnetic resonance imaging
MVD: Microvascular decompression
